# 

**Published:** 1886-12

**Authors:** 


### OUR ADVERTISERS.

FREDERICK STEARNS & CO.—This well-known house has a new advertisement in this number. Their goods rank among the best in the country. Try them.

REMOVAL.—Isaac Phillips, the instrument dealer, has removed from No. 13 N. Broad street to 14 Marietta street. He now has one of the handsomest places in the city, and is much better prepared to show goods than ever before. See his advertisement to be found on page 7.

HORSFORD'S ACID PHOSPHATE.—This preparation is very popular with every one, and bears the endorsement of many physicians in all parts of the country. If you have not used it, write to the Rumford Chemical Works, Providence, R. I., for a sample of it, and you will be pleased with it.

THANKS.—We have received from the Rio Chemical Company, of St. Louis, a package of the several preparations they manufacture. These goods are already well known to the profession, and are endorsed by some of the ablest men in the profession in this country. See their advertisement for a description of the preparations.

A NEW INSTRUMENT HOUSE FOR ATLANTA.—Chas. O. Tyner, the well-known druggist at No. 30 Marietta street, Atlanta, has an attractive advertisement in this issue of THE JOURNAL. Mr. Tyner says he intends to carry the most complete assortment of surgical instruments and appliances ever kept in this city, and that physicians can hereafter get anything they may need, either in the drug or instrument line, at his house, known to the trade. See his advertisement and write him for prices.

SYRUPUS ROBORANS.—This preparation of syrup of hypophosphites is manufactured by Arthur Peter & Co., of Louisville, who claim its flavor and perfection as a pharmaceutical compound; that it is an open and fair formula; that it is made accurately by that formula; that it is the cheapest to the trade, the physician and the consumer of any syrup hypophosphites compound now on the market; that it is a *perfectly stable syrup*. This stability makes it readily miscible with liquid pepsin, U. S. P. in from ⅓ to equal quantities, Fowler's solution in from 3 to 5 drops to the drachm and with syrup iodide iron, thus enabling you to unite the hypophosphites as a basis with these important agents to suit special cases.

